# Personalized Detection of Functional-State Changes Through Continuous Gait Monitoring: A Methodology for Assistive-Device Users

**DOI:** 10.3390/s26165234

**Published:** 2026-08-18

**Authors:** Janire Otamendi, Asier Zubizarreta, Imanol Torre, Cristina Sesma

**Affiliations:** 1 Department of Automatic Control and Systems Engineering, Bilbao School of Engineering, University of the Basque Country UPV/EHU, 48013 Bilbao, Spain; 2 Fekoor (The Coordinating Federation of People with Physical and/or Organic Disabilities in Bizkaia), 48014 Bilbao, Spain

**Keywords:** assistive-device users, gait anomaly detection, longitudinal study, one-class support vector machine, post-stroke patients

## Abstract

Lower limb mobility impairments resulting from neurological diseases, trauma injuries or aging significantly impact the quality of life of individuals by limiting their autonomy. Rehabilitation plays an important role in addressing the challenges of these impairments, with early detection of changes in the functional state of individuals being essential. This enables therapies to be adjusted based on the current condition of the patient, thereby enhancing their effectiveness. Such early detection, however, requires continuous assessment by specialists, which is unfeasible given the existing limited resources. Since gait is a reflection of the physical and mental states of each individual, its continuous monitoring and subsequent data analysis can serve as a valuable tool for the aforementioned objective. This study proposes a methodology that, based on continuous gait monitoring data, detects significant changes in the functional state of patients who require an assistive device for walking. Given the variability that may exist among different individuals, this methodology tackles the issue from an individualized approach generating personalized models for each individual using the OC-SVM technique. The proposed methodology was validated in nine healthy people who had different simulated functional states, obtaining an accuracy in the range of 70–97%. In addition, a one-year longitudinal study was also carried out with three post-stroke individuals to validate the methodology in real cases, obtaining an average accuracy of 78%.

## 1. Introduction

Lower limb mobility impairments affect millions of people worldwide and are expected to become increasingly prevalent due to population aging [1]. Beyond their impact on physical health, these impairments can significantly reduce autonomy, mental well-being, and overall quality of life. Rehabilitation plays a key role in improving functional capacity and mobility for these people [2,3]. However, effective therapy requires an accurate understanding of the patient’s current condition and its evolution over time, which, in practice, is difficult due to resource limitations and the growing number of individuals requiring care [4]. Furthermore, clinical evaluations are typically performed at isolated time points and under controlled conditions, making them dependent on the patient’s momentary state. Since gait is closely related to an individual’s physical and cognitive condition [5], its continuous monitoring represents a promising approach for functional assessment and the early detection of relevant changes over time.

Several technologies have been proposed in the literature for continuous gait monitoring, including wearable sensors [6,7], smartphones [8,9], and sensorized assistive devices for walking [10,11,12,13]. Wearable sensors are currently the most widely adopted solution, although they may cause discomfort as they must be attached to the body [14,15]. Smartphones are less intrusive but are often affected by parasitic movements that limit their applicability and precision. In contrast, sensorized assistive devices provide a non-invasive alternative for individuals who already require walking assistance in their daily lives, making them particularly suitable for the continuous monitoring of people with lower-limb motor impairments [10,11,12,13].

Supporting clinical decision-making using continuous gait monitoring, however, still presents significant challenges, as the acquired data must be properly analyzed to determine whether significant changes have occurred in the patient’s functional state. Most studies in the literature focus on the statistical analysis or classification of gait characteristics associated with specific pathologies [16,17]. While these approaches provide valuable clinical insights, the detection of functional changes still is not properly addressed in the literature, as it requires individualized modeling of each patient’s normal gait pattern and the identification of deviations from that baseline.

To address this problem, anomaly-detection techniques have been increasingly investigated [18,19,20,21]. Among them, machine learning approaches have attracted considerable attention because of their ability to model complex relationships and generalize across heterogeneous data [22]. In the context of gait anomaly detection, semi-supervised and unsupervised methods are particularly attractive because anomalous gait events are typically scarce and difficult to characterize comprehensively [23,24]. One-Class Support Vector Machines (OC-SVMs), in particular, have been widely employed for modeling normal gait behavior and identifying deviations that may reflect changes in functional status [25,26].

Although effective, most existing gait anomaly detection studies remain limited in their applicability. Many rely on controlled testing conditions, require specialist supervision, do not support continuous monitoring, or are not specifically designed for individuals who use assistive devices for walking. Furthermore, validation is frequently performed on healthy participants simulating impairments rather than on real-life patients, providing limited evidence regarding the feasibility of these approaches in real users.

To address these limitations, this work proposes a machine learning-based methodology for personalized gait anomaly detection using data acquired from a sensorized tip that can be attached to conventional walking aids [13]. The proposed framework is intended as a foundation for continuous functional-state monitoring in assistive-device users, enabling the identification of gait changes that may reflect clinically relevant functional alterations.

This study extends the methodology previously proposed by the authors in [27], which presented several limitations. In contrast to the previous approach, which relied on predefined anomalies, manual configuration procedures, and specialist intervention, the proposed framework supports individualized monitoring and autonomous detection of functional-state changes without requiring prior knowledge of the anomaly type.

The methodology is evaluated in two complementary settings. First, its ability to detect functional-state changes is assessed in a cohort of healthy participants under controlled conditions. Second, a preliminary longitudinal proof-of-concept evaluation is conducted with post-stroke individuals who use assistive devices for walking, providing an initial assessment of the feasibility of the approach in real users.

Thus, this work provides three main contributions with respect to previous works and the existing literature: (1) a personalized methodology for gait anomaly detection aimed at functional-state monitoring; (2) a framework specifically designed for assistive-device users based on a non-invasive sensorized tip, and (3) an experimental evaluation combining controlled testing in healthy participants with a preliminary longitudinal assessment in post-stroke individuals.

The rest of the article is organized as follows: Section 2 presents the sensorized tip used for continuous gait monitoring and data acquisition; Section 3 describes the experimental protocol and the procedures followed for data collection and dataset generation; Section 4 details the development of the gait anomaly detection methodology; Section 5 presents the results obtained in both healthy participants and post-stroke individuals; Section 6 discusses the main findings and limitations of the study; and finally, Section 7 summarizes the main conclusions and future research directions.

## 2. Sensorized Tip

**Figure 1:**
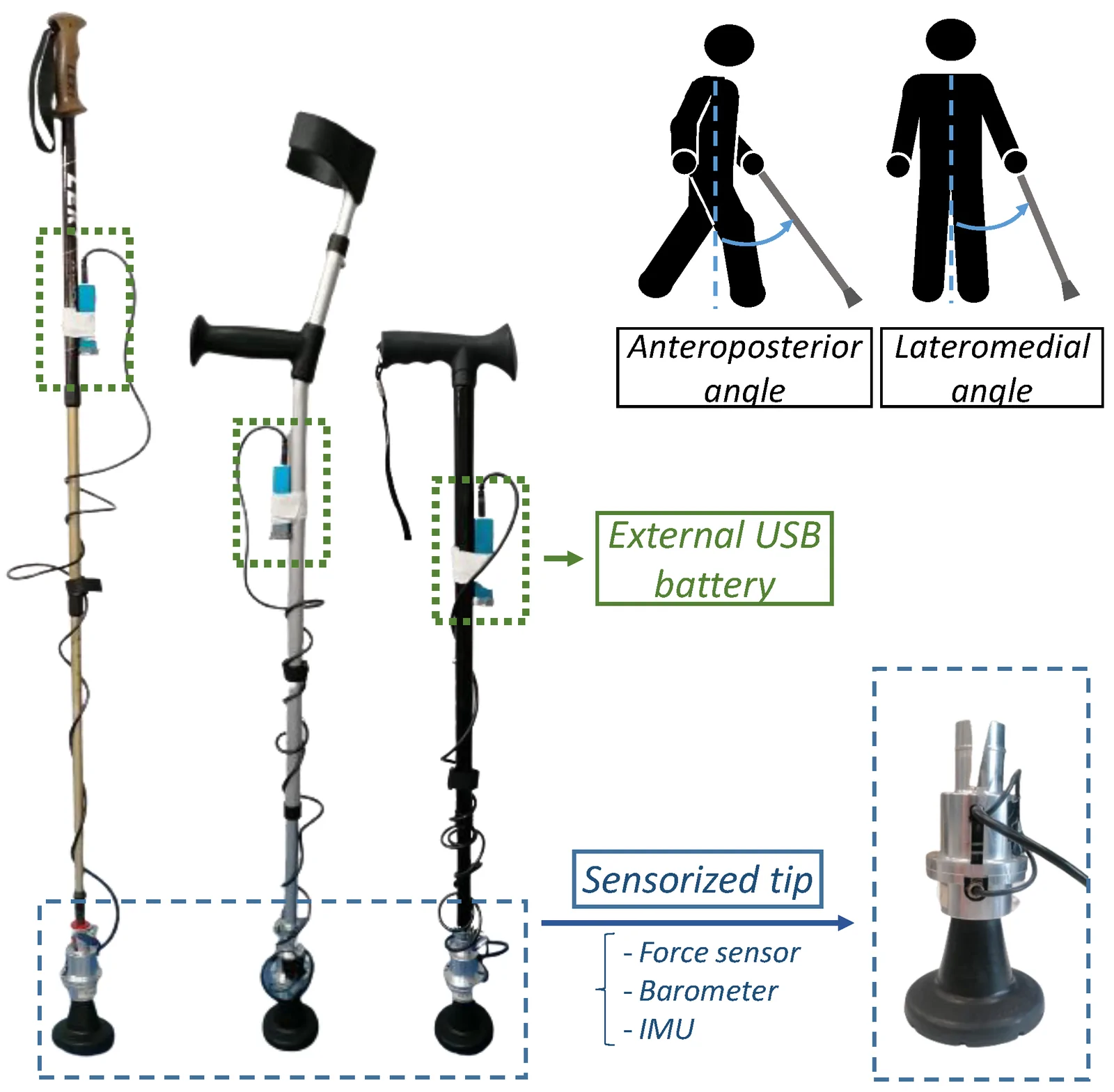
Sensorized tip, and a representation of the anteroposterior and lateromedial angles.

Given the advantages of using an assistive device for walking to monitor the gait of an individual, in this study, the sensorized tip presented in [13] is used (see Figure 1).

The main advantage of this device over other sensorized crutches or canes proposed in the literature is that it can be attached to the patient’s own assistive device. Thus, the patient does not have to adapt to a new one, making invasiveness minimal.

This sensorized tip integrates an acquisition system and three sensors (a force sensor, a barometer and an inertial measurement unit (IMU)), powered by an external standard USB-based battery. The force sensor provides the axial load exerted by the user; the barometer indicates the atmospheric pressure, which can be used to estimate the relative height of the device; and finally, the IMU captures the linear acceleration, angular speed and magnetic field in the local *x*, *y*, *z* axes of the crutch. This last sensor integrates also an algorithm that estimates the Euler angles in the global reference frame, which are then used to calculate the anteroposterior and lateromedial angles (see Figure 1). As detailed in [13], this device allows monitoring not only gait by means of the 3D motion of the device, but also the applied support force.

## 3. Experimental Protocol and Database Generation

To evaluate the proposed methodology, two complementary experimental studies were conducted. The first involved healthy participants performing controlled gait tests designed to emulate different functional conditions, while the second consisted of a longitudinal proof-of-concept study with post-stroke individuals. The data collected through these studies were used to generate the databases employed throughout the manuscript.

The following subsections describe the experimental protocols, data acquisition procedures, and database generation process for each study.

### 3.1. Experimental Trials with Healthy Participants

A series of tests were carried out by nine healthy volunteers in a controlled environment, with the approval of the Ethics Committee of the University of the Basque Country (UPV/EHU) (Code M10/2021/325).

It is important to note that these tests are not designed to mimic the evolution of post-stroke individuals. Instead, their aim is to provide a database to allow a controlled proof-of-concept evaluation of the proposed methodology to identify changes under different temporal configurations.

Given that the methodology has an individualized approach and generates personalized models, individuals of different ethnicities and genders were selected in order to provide a wider analysis in the validation process. Table 1 includes the main physical characteristics of each participant, including gender, body mass, height, and the side where the assistive device for walking (ADW) was used.

As the aim of the methodology is to detect significant changes in gait, its validation with healthy people requires these volunteers to simulate different gait patterns. For this purpose, various techniques are used in the literature: in some studies, participants are asked to dorsiflex and/or plantarflex their foot while walking [28], while in other works, knee movement is restricted using straps [21].

**Figure 2:**
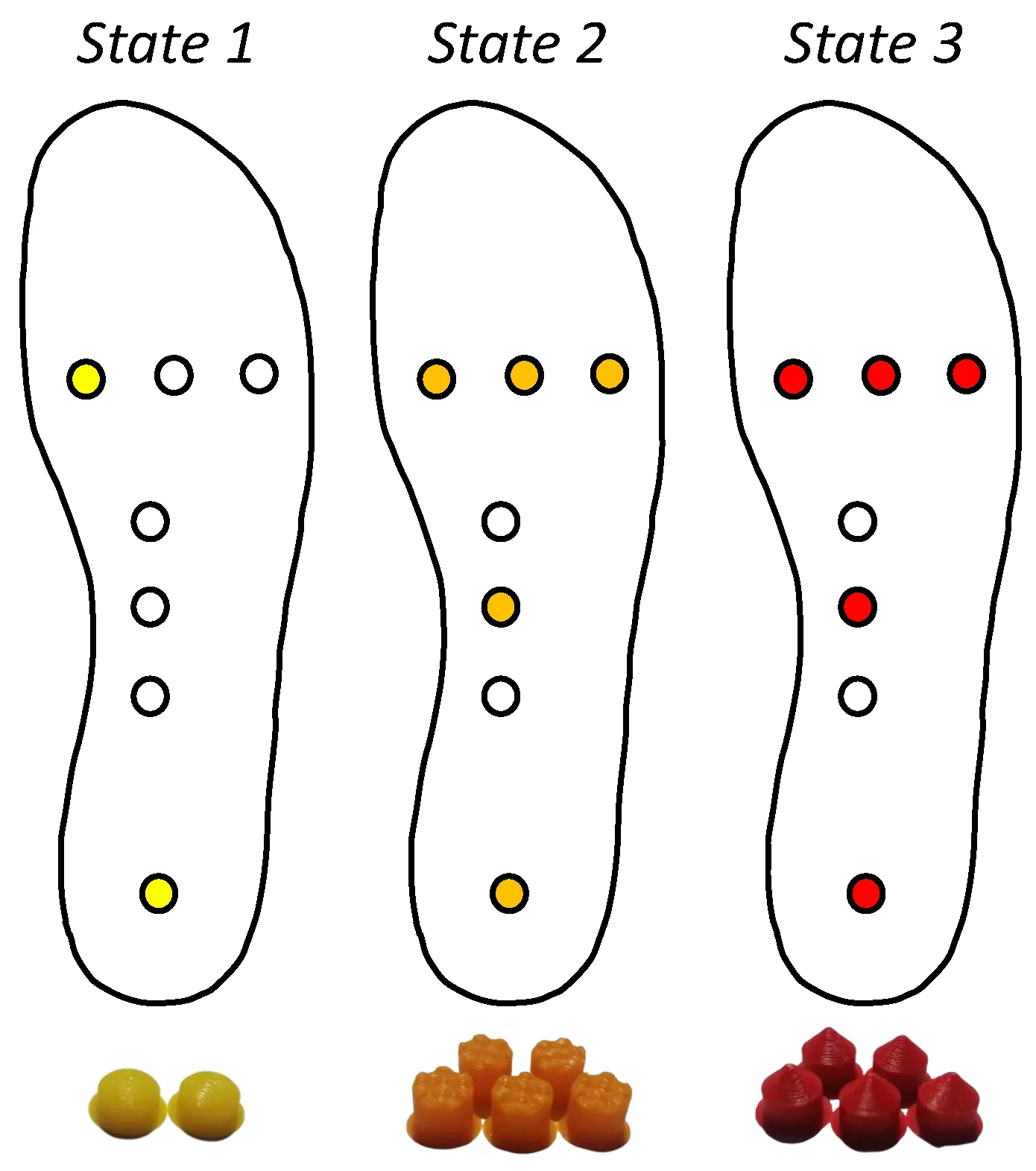
Impediments and their configuration for validation with healthy people.

The monitoring system to be used is a sensorized tip. Hence, the test design is focused on making the participants use an assistive device for walking out of necessity. For this purpose, the technique described in [27] is used, in which some 3D impediments are placed on the sole of the foot, causing discomfort and preventing the participant from walking without an assistive device. In order to simulate different functional states, three types of impediments are used (Figure 2). Each of them generates different degrees of discomfort due to its shape, which makes the gait pattern of each participant change according to the type of impediment used.

Note that the degree of discomfort generated by the impediments is different for each volunteer, as is the way in which each of them changes their gait pattern. The idea behind this technique is that this change is made unconsciously, thus making the validation with healthy people as similar as possible to trauma-related cases.

Based on this test design, in this work, the participants performed 5 tests with each type of impediment, i.e., 15 tests in total. Each test consisted of two repetitions in which volunteers walked 36 m in a straight line at a comfortable speed, using an assistive device for walking with the sensorized tip attached. In order to capture the variability that may exist within the same functional state, these tests were performed on different days. During all tests, the sensor data detailed in Section 2 were captured and labeled considering the participant and impediments used for the test. Across all tests, this protocol yielded approximately 760 analysis windows, i.e., swing–stance cycles of the crutch, per healthy participant, with similar values observed across individuals.

Once the test data was captured, the aforementioned tests were ordered with respect to the simulated time-evolution of a particular participant. That is, the tests were ordered to generate a set of different time-sequences in order to emulate multiple temporal sequences containing different combinations of transient and persistent changes, allowing the behavior of the proposed methodology to be evaluated under a variety of controlled scenarios. For this purpose, 100 different time-sequences were randomly generated for each participant, and this process was repeated 10 times to reduce potential biases associated with a particular ordering.

Considering 15 tests corresponding to 3 simulated states (5 per state), the generation of each time-sequence is conducted as follows:

1. A state is randomly chosen.
2. n tests corresponding to the selected state are chosen randomly, with 2≤n≤N, where *N* is the number of remaining tests corresponding to the selected state. Note that at least two test for the same initial state are selected in order to ensure proper characterization of the initial state, as later analyzed in the proposed methodology.
3. We return to the first step by removing the tests that have already been used.

This procedure is repeated until the 15 tests are arranged in each of the time-sequences, generating the overall database which was used to test the state change detection ability of the methodology, detailed later, under controlled conditions.

### 3.2. Experimental Trials with Post-Stroke Individuals

The second set of tests is focused on post-stroke individuals who require assistive devices for walking, with the aim of providing a dataset in order to perform a proof-of-concept study of the gait anomaly detection capabilities of the methodology proposed in this work. For this purpose, a longitudinal pilot study was carried out in the non-profit organization Fekoor, the Coordinating Federation of People with Physical and/or Organic Disabilities in Bizkaia, with the approval of the Ethics Committee of the University of the Basque Country (UPV/EHU) (Code M10/2022/334).

Given that the monitoring system to be used is the sensorized tip presented in Section 2, three post-stroke volunteers who use an assistive device for walking in their daily life were recruited for this pilot longitudinal study. Table 2 summarizes their main data, including gender, body mass, height, and the side where the assistive device for walking (ADW) is used.

Although the proposed methodology is intended for continuous operation, this proof-of-concept study was conducted through weekly assessment sessions under therapist supervision. This design enabled proof-of-concept validation while ensuring accurate identification and labeling of functional-state changes within a controlled monitoring protocol.

Each weekly assessment session consisted of three stages: (1) a pre-test evaluation, (2) a gait monitoring test, and (3) a post-test evaluation. During the pre- and post-test assessments, therapists gathered information regarding the participant’s functional condition using standard clinical evaluations and patient-reported questionnaires. These assessments were intended to support the interpretation of the participant’s status and evolution over time. The gait monitoring phase consisted of two repetitions of a 19 m straight-line walking test performed at a comfortable pace using the volunteer’s own assistive walking device equipped with the sensorized tip.

Two rehabilitation specialists from Fekoor participated in the study. Each volunteer was assigned to a single reference specialist, who was responsible for their regular clinical follow-up and therefore had direct knowledge of their functional evolution throughout the study period. Based on the information collected during the assessments and their clinical judgment, the specialist determined whether a relevant functional-state change had occurred. The specialist also classified the observed changes according to their nature and persistence, distinguishing between transient and permanent changes.

The labels used in this study were defined exclusively according to the specialists’ assessment. The remaining clinical measurements and questionnaires were collected to provide contextual information regarding the participants’ condition and to facilitate the interpretation of the results.

Across all sessions, this protocol resulted in approximately 2550 analysis windows (swing–stance cycles of the crutch) for the first volunteer, 1600 for the second, and 720 for the third. This data was included in a database used to perform a proof-of-concept evaluation of the performance of the gait anomaly detection methodology proposed in the next section.

## 4. Gait Anomaly Detection Methodology for Continuous Monitoring

The aim of the proposed methodology is to identify anomalies in the functional state of an individual based on continuous gait monitoring data such as those captured in the aforementioned tests. Although the development in this section considers the information provided by the sensorized tip presented in Section 2, the proposed methodology can also be adapted to other gait monitoring systems.

It is important to note that, while the approach relies on continuous monitoring, the methodology outlined in this study specifically utilizes data captured during instances when the user is walking in a straight line at a comfortable speed. This physical activity can be readily identified with the assistance of an activity classifier, such as the one proposed in [29]. Moreover, as individuals may present variability in their condition, this methodology adopts an individualized approach, generating personalized models derived from each individual’s data.

**Figure 3:**
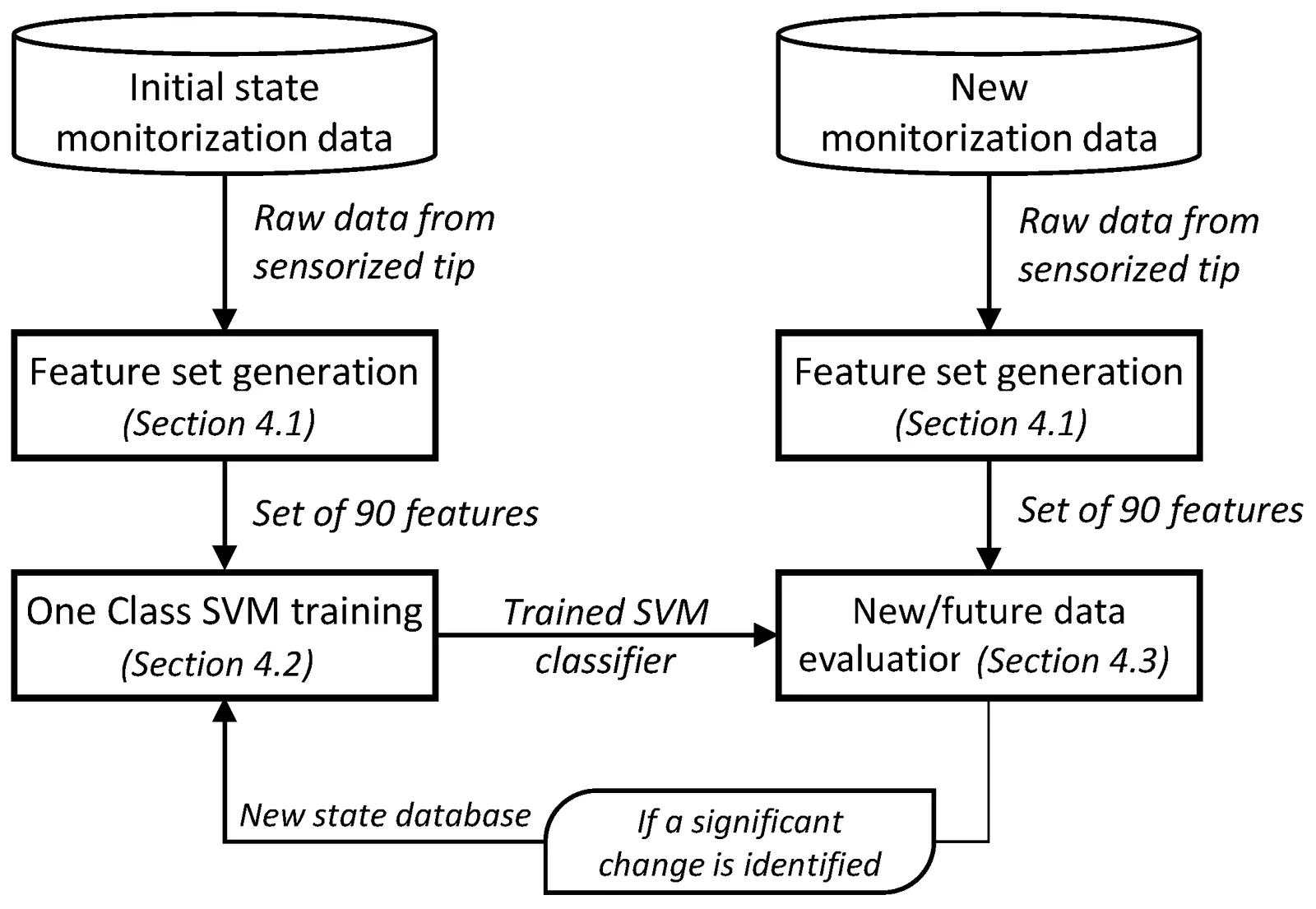
Summary of the gait anomaly detection methodology for continuous monitoring.

The proposed approach, based on the literature concerning anomaly detection and pattern recognition [22], is summarized in Figure 3, briefly described below, and explained in detail in subsequent subsections.

In order to detect an anomaly, characterizing the current state of the individual or patient is required first. Although some studies work directly with raw captured data [30,31], this study employs a feature engineering-based approach. Widely used in the field of machine learning, this method facilitates data handling and simplifies the problem. Hence, the initial step of the proposed methodology consists of processing the raw captured data to generate a set of representative features, which will serve as the input for the following stages (Section 4.1).

Secondly, using the current state characterization data, an individualized model is trained, by means of a Support Vector Machine (SVM) (Section 4.2). As detailed in Section 1, this technique is one of the most widely used in the field of anomaly detection and gait recognition, given its ability to generalize even on small datasets [22,32].

Finally, once the model is trained, it is used to evaluate the data corresponding to subsequent time instants, identifying whether they belong to the same group (normal state) or not (anomaly). If no persistent anomaly is detected, the detector will continue to evaluate new input data. However, if a persistent anomaly is detected, a change in the functional state of the individual will be assumed, and a new model will be generated from these data (Section 4.3).

### 4.1. Feature Set Generation

Feature-based engineering is used in this study to process the raw data and generate a set of features that could be relevant for gait anomaly detection. This process involves two main phases: segmentation and characterization.

**Figure 4:**
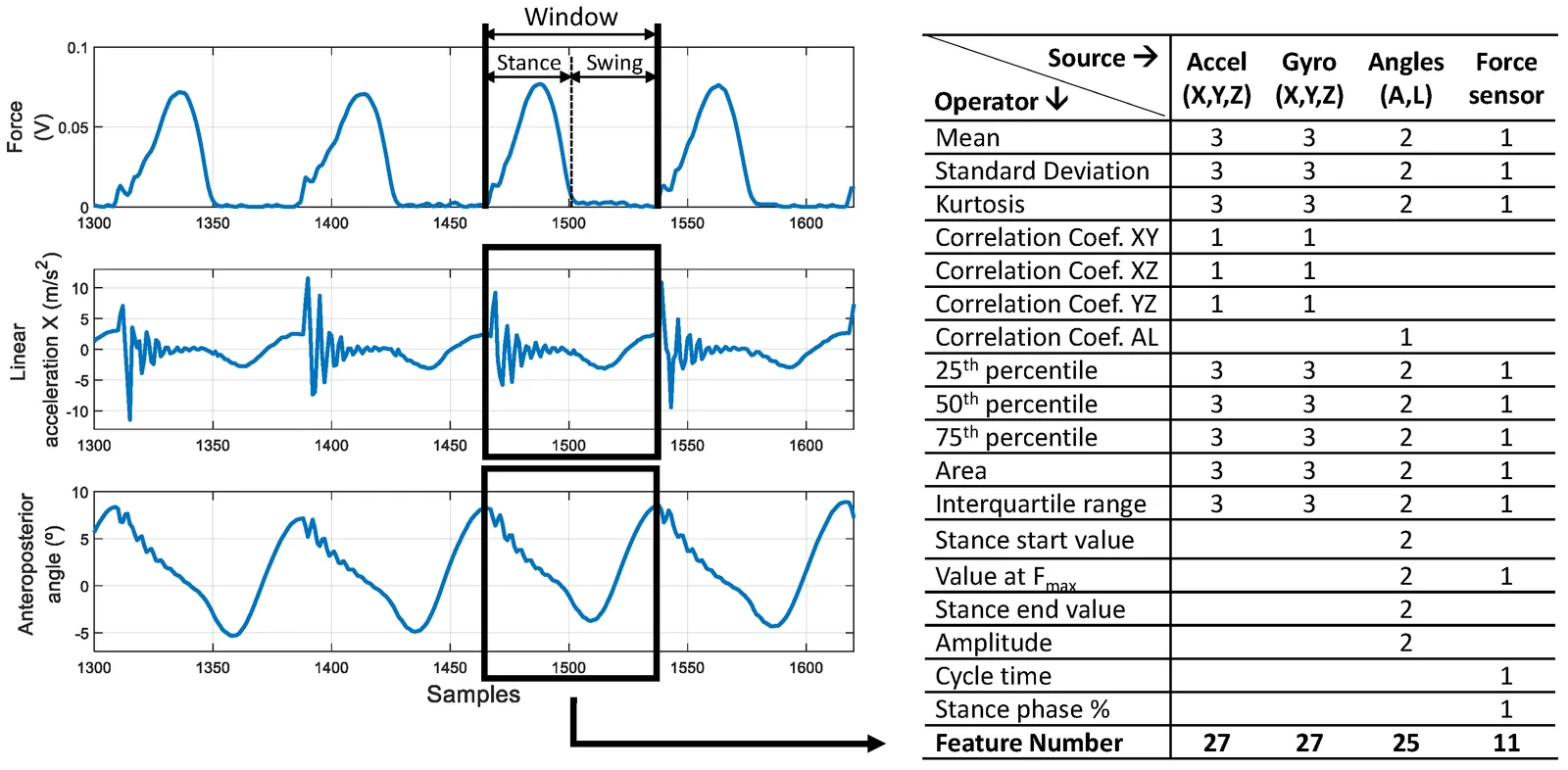
Feature set generation procedure: segmentation and characterization phases.

During the segmentation phase, the signals are divided into sequential analysis windows associated with the cycles of use of the assistive device for walking (ADW). Each of these cycles comprises a stance phase and a swing phase, which are easily discernible by the force signal recorded by the sensorized tip (see Figure 4). The beginning of each window is determined by the moment when the tip makes contact with the ground, thereby delineating the segmentation process.

The subsequent characterization phase consists of extracting features from each discrete window obtained during the segmentation phase. For this purpose, and based on the proposal presented in [29], a set of statistical operators is applied to the signals provided by the sensorized tip, yielding a series of features that characterize each cycle of use of the crutch for subsequent gait anomaly detection. Figure 4 summarizes the features generated during this data processing phase. In this Figure, Accel refers to the accelerometer data and Gyro to the gyroscope data, with X, Y and Z representing the axes related to the acceleration or angular velocity measurement. Angles, on the other hand, refer to the estimated anteroposterior (A) and lateromedial (L) angles, as indicated in Figure 1.

Note that in this case, the magnetometer and Euler angle signals have not been used, since they deal with absolute angles that can lead to errors. Similarly, the barometer signal has not been used either, as its relative accuracy (0.12 hPa ≈ 1 m) is not sufficient to capture height variations in a straight-line walking test.

After performing the two phases of data processing, a total of 90 features are obtained for each of the analysis windows.

### 4.2. One-Class SVM Training

After monitoring the patient’s gait corresponding to their current state and generating features using the procedure described in Section 4.1, a One-Class Support Vector Machine (OC-SVM) classifier is trained.

The OC-SVM is a semi-supervised machine learning technique trained using data from a single class (the normal or reference class). Subsequently, it classifies new data as belonging to the same class or as different, making it particularly useful for anomaly detection applications, including the gait analysis field.

In this study, the OC-SVM model is implemented using the ocsvm function from MATLAB’s r2024b Statistics and Machine Learning Toolbox. A radial basis function (RBF) kernel is employed, as it allows the model to capture non-linear decision boundaries in the feature space. This implementation automatically tunes key hyperparameters, such as the kernel scale and regularization parameter, based on the characteristics of the training data. Consequently, the only parameter that requires explicit definition is the contamination fraction, which represents the expected proportion of anomalous samples within the training dataset.

The value of the contamination fraction depends on the expected proportion of anomalous samples within the training dataset and therefore on the characteristics of the monitored population. As an initial approach, a value of 0.1 can be considered, allowing for the presence of a limited number of anomalous samples in the training set while ensuring that anomalies remain rare events [33]. Depending on the variability of the data and the application scenario, this parameter may be further refined through experimental analysis. Since the optimal value can differ across populations and individuals, its selection should be adapted to the specific deployment context, as it will be analyzed for the datasets considered in this work.

The methodology is based on an individualized approach, with a separate OC-SVM model trained for each participant using only their own data, establishing a model that characterizes the participant’s current state and serves as the basis for anomaly detection. Therefore, it is vital to ensure that the training data accurately represent the participant’s state without introducing excessive variability, as that could hinder anomaly detection. In the datasets considered in this study, employing data from two tests (straight walking) conducted on different days was found to provide a suitable balance between representativeness and consistency when characterizing the participant’s baseline state.

### 4.3. New/Future Data Evaluation

**Figure 5:**
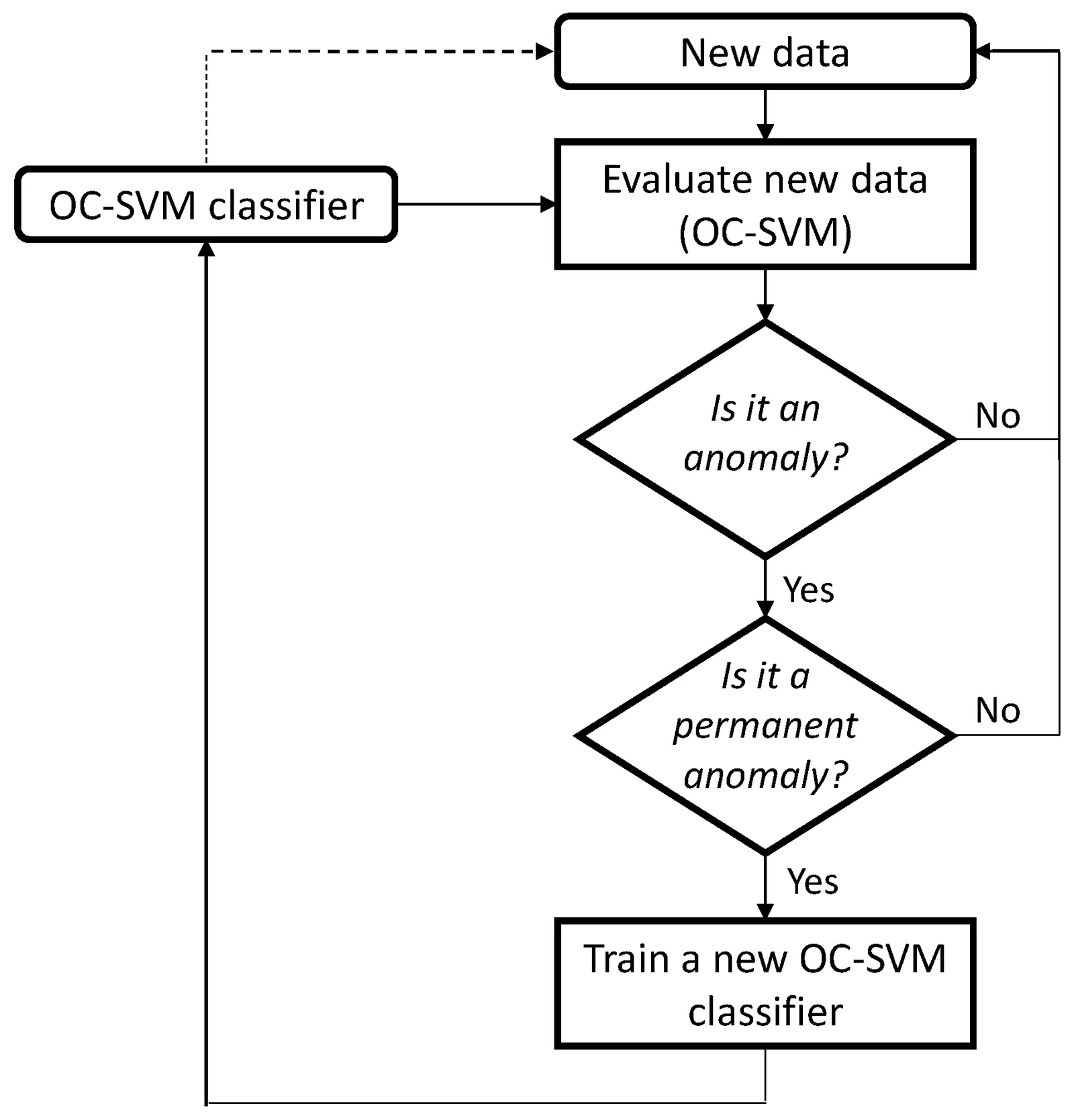
New data evaluation process and continuous operation of the methodology.

Figure 5 summarizes the evaluation process, along with the continuous operation of the methodology.

First, the OC-SVM classifier trained through the procedure described in Section 4.2 is used to evaluate new data. Each monitoring session is segmented, considering the assistive device for walking cycles, into multiple analysis windows, which are independently classified as either belonging to the normal state (the one learned by the system) or as anomalous. Once all windows within a session have been evaluated, a session-level decision is obtained through a majority voting scheme. Specifically, the number of analysis windows classified as anomalous is compared with the number classified as normal, and the session is labeled as anomalous only when the majority of its analysis windows are classified as anomalous. Otherwise, the session is considered to correspond to the normal state.

If the monitoring session is not considered anomalous by the previous approach, it is considered that gait is similar to the normal model learned by the OC-SVM. In this case, the individualized model that characterizes the patient’s current state, implemented in the OC-SVM classifier, is maintained as the reference model for future evaluations. Note that continuously updating the individualized model may lead to classification errors, as small but progressive variations in the functional state of the patient may be incorporated into the model over time. As a consequence, substantial changes occurring in the medium or long term could become increasingly difficult to detect.

On the other hand, the detection of an anomaly does not necessarily imply that a relevant functional-state change has occurred. Gait can be temporarily affected by factors such as fatigue, discomfort, lack of attention, or other transient circumstances. Therefore, to reduce the influence of isolated events, the proposed methodology considers that a substantial functional-state change has occurred only when anomalies are detected in two consecutive monitoring sessions. This conservative decision rule is intended to increase the robustness of the system and prevent unnecessary updates of the individualized model due to short-term fluctuations.

When this condition is satisfied, the detected change is assumed to represent a lasting change in the participant’s functional state, and a new OC-SVM classifier is trained using the anomalous data to characterize the new state. Otherwise, the anomaly is considered temporary, and the individualized model remains unchanged for future evaluations.

When a significant functional-state change is detected, a specialist should assess the patient and adjust their rehabilitation accordingly. However, if resource constraints prevent immediate assessment, the system will continue operating, maintaining a log of both data and detected anomalies, so that this information can be provided to the specialist when required.

## 5. Results

In this section, the results obtained by applying the methodology proposed in this work to the datasets generated in Section 3 for both healthy participants and post-stroke individuals are detailed. For evaluation purposes, each monitoring session is assessed independently with respect to the functional state represented by the individualized model available at that time. Sessions that differ from the modeled state are considered positive instances, whereas sessions consistent with the modeled state are considered negative instances. Accuracy, sensitivity, specificity, and precision are computed from the resulting numbers of true positives (TPs), true negatives (TNs), false positives (FPs), and false negatives (FNs).

### 5.1. Healthy Participants Dataset Results

**Figure 6:**
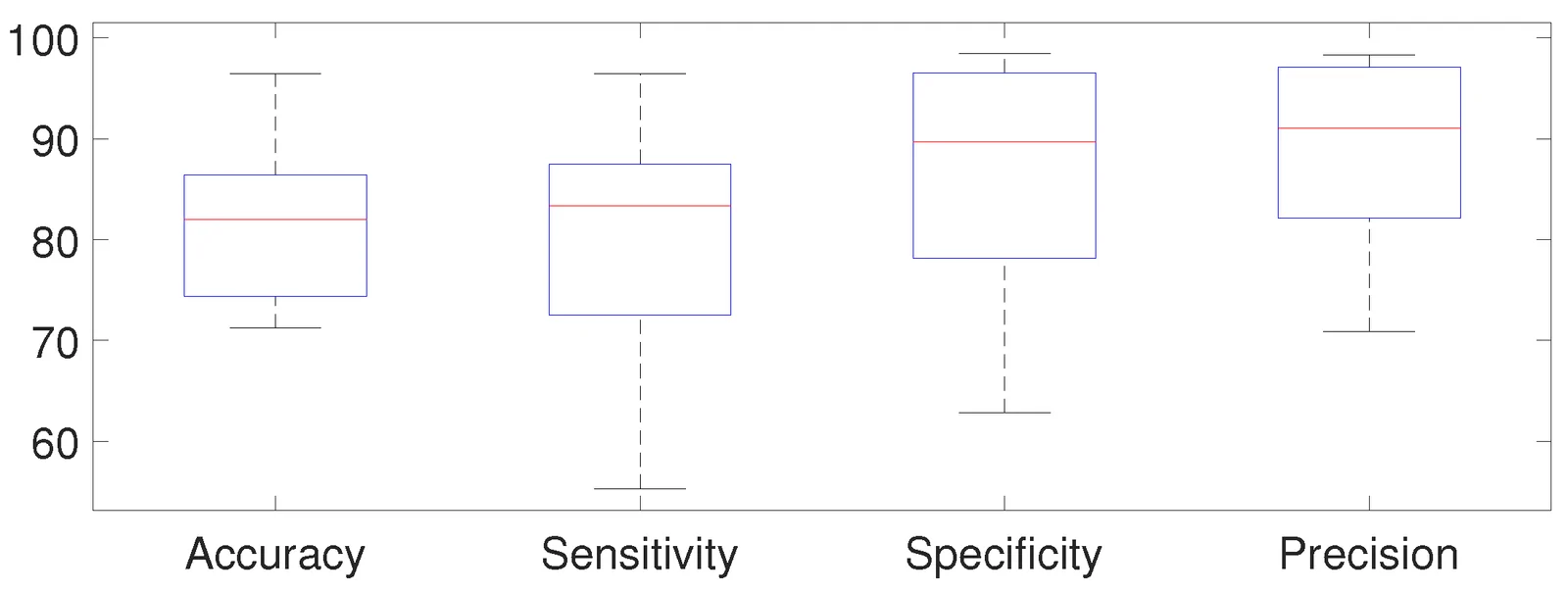
Distribution of performance metrics across healthy participants.

Table 3 reports the total of true positives (TP), true negatives (TN), false positives (FP), and false negatives (FN) per volunteer, while Table 4 summarizes the performance of the proposed methodology across the nine healthy volunteers, reporting accuracy, sensitivity, specificity, and precision. Figure 6 shows the inter-subject variability of these metrics using a box plot, in which the red line shows the mean, while the upper and lower blue lines represent the 25th and 75th percentiles.

### 5.2. Post-Stroke Individuals Dataset Results

**Figure 7:**
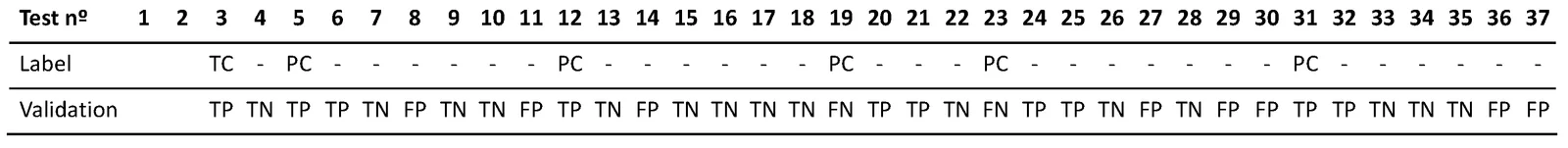
Participant 1 testing: specialist feedback and methodology results (TC = transient change, PC = permanent change, TP = true positive, TN = true negative, FP = false positive, FN = false negative).

**Figure 8:**
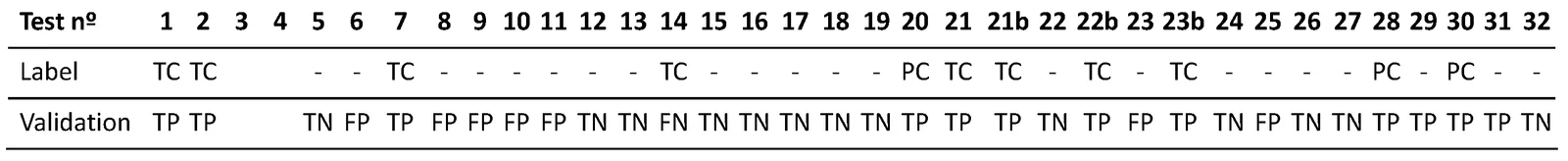
Participant 2 testing: specialist feedback and methodology results (TC = transient change, PC = permanent change, TP = true positive, TN = true negative, FP = false positive, FN = false negative).

**Figure 9:**
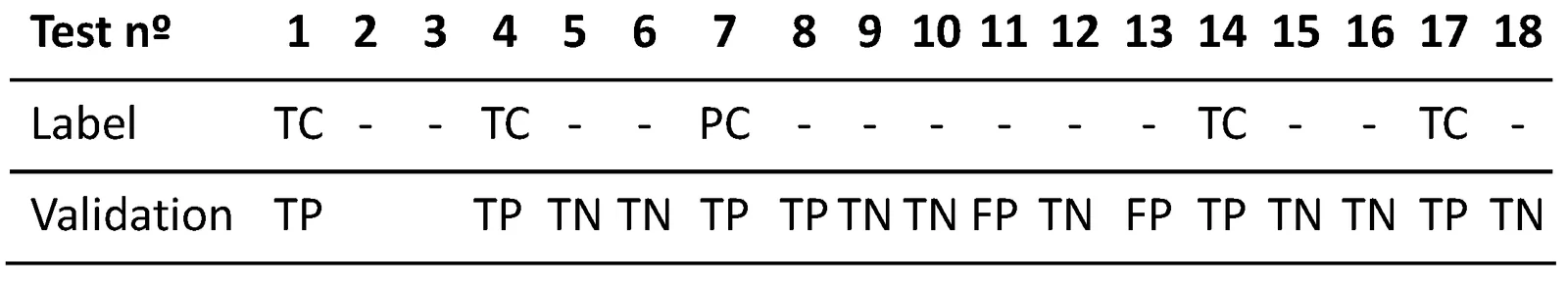
Participant 3 testing: specialist feedback and methodology results (TC = transient change, PC = permanent change, TP = true positive, TN = true negative, FP = false positive).

For the post-stroke individuals dataset, Table 5 reports the performance metrics obtained for each participant, while Table 6 provides the corresponding numbers of true positives (TPs), true negatives (TNs), false positives (FPs), and false negatives (FNs). Figure 7, Figure 8 and Figure 9 show the corresponding session-level outcomes. These figures compare the labels provided by the specialists with the predictions of the proposed methodology, including true positives, true negatives, false positives, and false negatives.

As described in Section 4, two initial sessions are used to characterize the baseline state of each participant. For participants 2 and 3, these initial sessions were not fully representative of their normal condition due to external factors (use of different assistive devices or non-representative walking conditions). In these cases, alternative sessions were selected for model initialization and validation, as detailed in the corresponding figures.

Each session is evaluated independently using the model corresponding to the participant’s current state. Table 5 provides a summary of the quantitative results for all post-stroke participants, showing accuracy ranges from 71.4% to 87.5%, with variations in sensitivity, specificity, and precision across subjects.

## 6. Discussion

The results obtained for both the controlled healthy volunteer and real-world post-stroke individual datasets are discussed next in order to verify the potential of the proposed approach for this pilot study.

In this discussion, it is important to emphasize that the proposed approach identifies deviations from a participant-specific reference functional state. Hence, a detected deviation is classified as a positive instance; however, only persistent deviations across monitoring sessions lead to a model update and are interpreted as a functional-state change. Isolated anomalous detections do not trigger any adaptation of the model and are not considered indicative of a change in functional state.

### 6.1. Healthy Volunteers

The results show that for the healthy volunteers dataset, an average accuracy of 82% was obtained, which is comparable to results reported in previous studies using wearable sensor-based anomaly detection approaches [21,34]. Variability across participants was observed, with accuracy values ranging from 71% to 96%. This variability is mainly related to differences in within-state consistency and the separability between simulated gait conditions.

**Figure 10:**
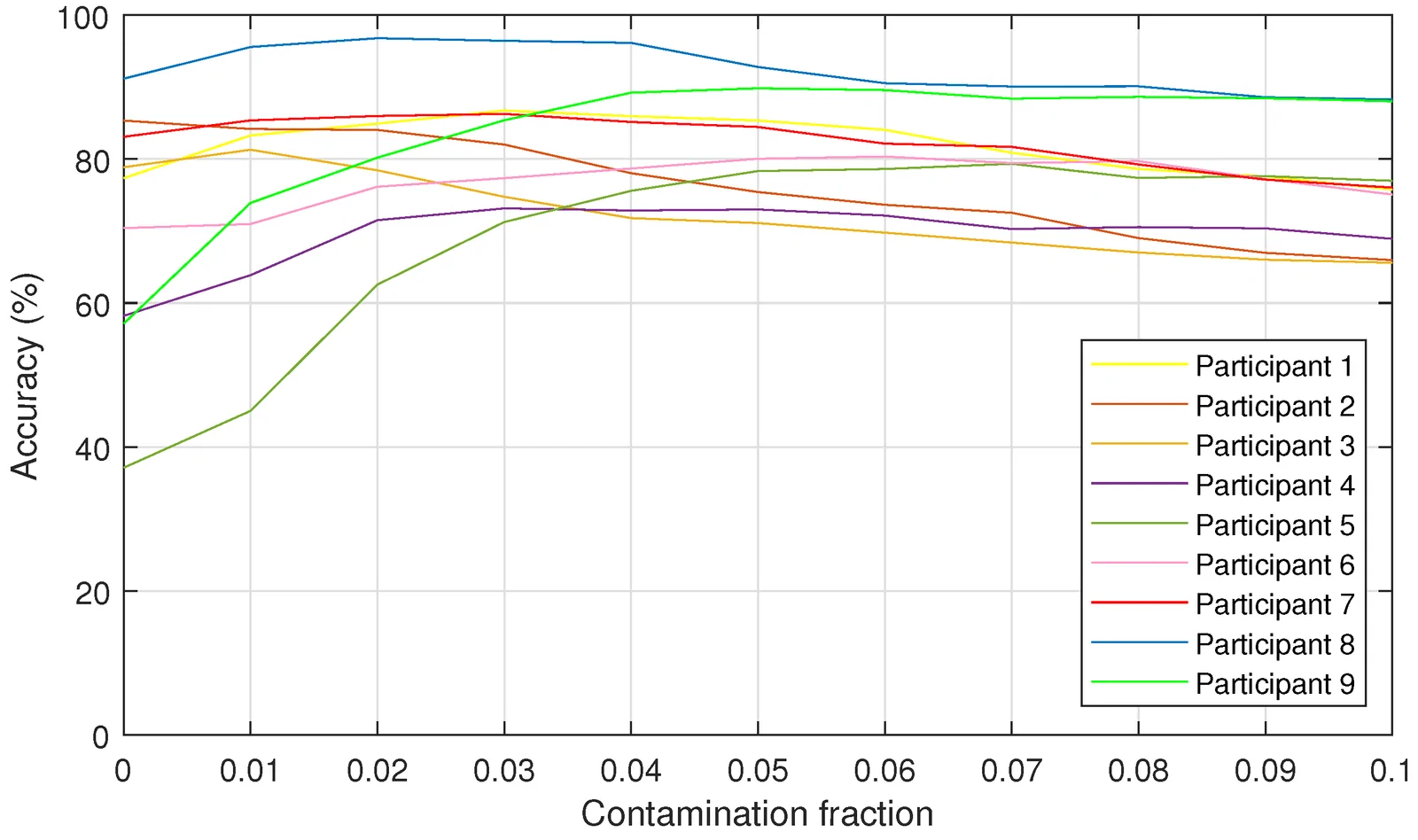
Accuracy according to the contamination fraction.

The influence of the contamination fraction was also analyzed (Figure 10). In the present study, an initial contamination fraction of 0.1 was considered for all healthy participants, based on the assumption of a low proportion of anomalous samples in the training data [33]. An exploratory analysis conducted on three randomly selected participants showed that a lower value of 0.03 led to improved performance, which was consistent with the highly controlled nature of the experimental conditions. This value was subsequently applied to the remaining participants to maintain consistency across the cohort. The sensitivity analysis indicates that, although classification performance varies with the selected contamination fraction, the proposed methodology remains relatively stable across a broad range of values. These results suggest that the approach is reasonably robust to moderate variations in this hyperparameter, while also indicating that participant-specific tuning may further improve performance in individualized monitoring scenarios.

### 6.2. Post-Stroke Individuals

For the post-stroke individuals dataset, the scenario is more complex due to the nature of the data. In case of participant 1, two false negatives were identified in sessions 19 and 23. Both correspond to sessions where an external element was introduced, the knee brace in session 19 and the new DAFO in session 23. It must be taken into account that these devices were implemented shortly before the test, and therefore, the participant did not have time to get used to them. As such, it was observed that including these devices did not present noticeable effects immediately during the first session. However, after several days and proper training with therapists on the use of the new device, its effect became evident in gait patterns. This was indeed the case in this study, where the methodology failed to detect the change in the first session, but successfully identified it in the subsequent one.

On the other hand, eight false positives are also identified for participant 1, which results in a precision of 55.6% for this individual. Most of these correspond to isolated anomalies, except for sessions 29–30 and 36–37, where the system erroneously identifies consecutive anomalies. It is worth noting that the validation relies on labels provided by specialists through an observation-based assessment, which means that subtle variations not apparent to clinicians may not be reflected in the labeling. Consequently, some subtle gait variations may not be fully represented in the available labels. A representative example is session 11, where the system reported an anomaly that was not identified by the specialists; interestingly, the participant experienced a fall shortly after this session. Although no causal relationship can be established, this observation illustrates the difficulty of validating subtle gait variations that may not be reflected in the available clinical labels and the potential of the proposed methodology to capture early or subtle gait variations. However, performing a robust validation of such detections poses a significant challenge, particularly regarding labeling, since changes that clinicians cannot observe cannot be annotated for traditional validation.

From a practical perspective, false positives must be carefully considered, as excessive false alarms could reduce the usefulness of a monitoring system. However, in the proposed methodology, an isolated anomalous detection does not trigger any model update or clinical action. A permanent change is only considered when anomalies are detected in two consecutive monitoring sessions, which reduces the influence of occasional false positives caused by day-to-day variability. As a result, the system is designed to prioritize persistent deviations from the reference state rather than isolated events. This mechanism helps limit the practical impact of sporadic false alarms while maintaining sensitivity to meaningful changes in gait patterns.

In the case of participant 2, a single false negative is identified, corresponding to a test where the foot-up was used instead of the DAFO. Several false positives are also observed, especially in the initial tests; however, it is worth noting that in session 7, there was a stumble, which was treated as a punctual anomaly in terms of labeling, but there may have been unidentified changes in the assessments conducted during those sessions. Once these variations stabilize, the system’s performance accuracy significantly improves.

Finally, in the case of participant 3, two false positives are observed, while the remaining sessions are correctly identified. This participant took part in fewer sessions compared to participants 1 and 2; therefore, the results are not directly comparable to those of the rest of the participants.

### 6.3. Comparison Between Datasets

Comparing the two datasets—healthy participants and post-stroke individuals—highlights the distinct challenges posed by each group. Healthy participants performed their tests in a controlled and conscious manner, with clearly differentiated simulated states and a lower likelihood of error. Conversely, real patient scenarios were inherently more variable. Post-stroke individuals walked as they would in their daily lives, introducing factors such as accumulated fatigue, occasional distractions, or fluctuations in effort during the test. This variability led to more anomalous steps and less consistency in samples within the same state. Furthermore, even if the study was carried out in a controlled environment, transitions between states were not as clearly defined, posing challenges even for specialists. These differences highlight the challenges associated with longitudinal gait monitoring in real-world rehabilitation settings and support the use of objective monitoring methodologies for tracking functional changes over time.

Overall, these results indicate that the proposed methodology presents potential to identify individualized gait state deviations in both controlled and real-world conditions. While performance is influenced by participant-specific variability, the longitudinal framework successfully detected functional changes across different scenarios, supporting its potential as an objective tool for gait monitoring.

### 6.4. Limitations

This study presents several limitations that should be considered when interpreting the results. First, the longitudinal validation in post-stroke participants included only three individuals. Although repeated measurements allowed the evaluation of functional-state changes over several months within each participant, the sample size is insufficient to support broad conclusions regarding population-level generalizability. Therefore, the post-stroke evaluation should be regarded as a preliminary longitudinal assessment aimed at investigating the feasibility and potential of the proposed methodology in real users of assistive devices.

Second, while the proposed framework is intended for continuous gait monitoring in real-world settings, the present study evaluated its performance through controlled assessments. This approach was adopted as an initial step to establish the methodological foundations and investigate the capability of the system to detect individualized functional-state changes under known conditions. Future studies should involve larger cohorts and long-term real-world deployments to further evaluate robustness, ecological validity, and clinical utility.

Third, the contamination fraction was selected empirically and kept fixed within each cohort. Although the sensitivity analysis suggests reasonable robustness, participant-specific tuning may further improve performance.

Finally, the evaluation in post-stroke participants relies on therapist-based annotations of functional-state changes, which may not fully capture subtle or transitional variations in gait patterns. This introduces a limitation related to the availability and granularity of reference annotations, as some changes identified by the proposed system may not be reflected in the clinical labeling due to the inherent subjectivity and observational nature of the assessment process.

## 7. Conclusions

This study presents a ML-based novel methodology to detect changes in the functional state of individuals through continuous gait monitoring. The primary objective is to promptly identify alterations, enabling healthcare specialists to intervene in a timely manner and adjust therapy to individual condition. The methodology relies on data collected from a sensorized tip, making it applicable to individuals that use assistive devices for walking in their daily lives.

The proposed approach was evaluated in cohorts of healthy individuals and post-stroke individuals with hemiplegia, achieving average accuracies of 82% and 78%, respectively. While the results obtained in healthy participants support the capability of the methodology to detect functional-state changes, the post-stroke evaluation should be considered a preliminary longitudinal validation due to the limited number of participants. Nevertheless, the findings provide initial evidence of the feasibility and potential of the proposed approach in real users of assistive devices.

This work contributes to the literature by introducing a personalized framework for functional-state monitoring and providing evidence of its capability to detect functional gait changes in both healthy and post-stroke individuals. The proposed methodology represents a promising step toward continuous assessment of assistive-device users, with the potential to support early identification of functional deterioration and facilitate more timely and personalized clinical interventions.

The findings also underscore the importance of accurately characterizing the individual’s state. Therefore, future endeavors will focus on refining parameters such as the number of tests used for characterization and the contamination fraction, both of which have shown efficacy in improving results.

## Figures and Tables

**Table 1:** Main data of healthy volunteers.

	Gender	Body Mass (kg)	Height (cm)	ADW Side
Participant 1	Male	84	184	Right
Participant 2	Male	63	185	Right
Participant 3	Female	63	163	Left
Participant 4	Male	75	180	Right
Participant 5	Female	67	167	Right
Participant 6	Male	76	179	Left
Participant 7	Male	79	178	Right
Participant 8	Female	69	165	Left
Participant 9	Female	50	158	Left

**Table 2:** Main data of post-stroke volunteers.

	Gender	Body Mass (kg)	Height (cm)	ADW Side
Participant 1	Female	53	153	Right
Participant 2	Male	85	181	Left
Participant 3	Male	72	169	Left

**Table 3:** Summary of classification outcomes (TP, TN, FP, FN) for healthy participants.

	TP	TN	FP	FN
Participant 1	6347	4931	565	1157
Participant 2	6201	4461	1775	563
Participant 3	5730	3989	2357	924
Participant 4	5053	4456	763	2728
Participant 5	4533	4730	12	3660
Participant 6	5568	4487	1096	1849
Participant 7	5987	5230	588	1195
Participant 8	6992	5545	203	260
Participant 9	5951	5151	182	1716

**Table 4:** Summary of the performance metrics for the healthy cohort.

	Accuracy [%]	Sensitivity [%]	Specificity [%]	Precision [%]
Participant 1	86.8	84.5	89.7	91.8
Participant 2	82.0	91.7	71.6	77.8
Participant 3	74.8	86.1	62.9	70.9
Participant 4	73.1	64.9	85.4	86.9
Participant 5	71.3	55.3	98.4	98.3
Participant 6	77.3	75.1	80.4	83.6
Participant 7	86.3	83.3	89.9	91.0
Participant 8	96.4	96.4	96.5	97.2
Participant 9	85.4	77.6	96.6	97.0

**Table 5:** Summary of the performance metrics for the post-stroke cohort.

	Accuracy [%]	Sensitivity [%]	Specificity [%]	Precision [%]
Participant 1	71.4	83.3	65.2	55.6
Participant 2	75.8	92.3	65.0	63.2
Participant 3	87.5	100	80.0	75.0

**Table 6:** Summary of classification outcomes (TP, TN, FP, FN) for post-stroke participants.

	TP	TN	FP	FN
Participant 1	10	15	8	2
Participant 2	12	13	7	1
Participant 3	6	8	2	0

## Data Availability

Data is unavailable due to privacy or ethical restrictions.

## References

[1] Gimigliano F., Negrini S. (2017). The World Health Organization “Rehabilitation 2030: A call for action”. Eur. J. Phys. Rehabil. Med..

[2] Heinemann A.W., Feuerstein M., Frontera W.R., Gard S.A., Kaminsky L.A., Negrini S., Richards L.G., Vallée C. (2020). Rehabilitation Is a Global Health Priority. BMC Health Serv. Res..

[3] World Health Organization (2017). Violence and Injury Prevention. Rehabilitation in Health Systems.

[4] Flachenecker P. (2015). Clinical implications of neuroplasticity - the role of rehabilitation in multiple sclerosis. Front. Neurol..

[5] Nagano H., Sarashina E., Sparrow W., Mizukami K., Begg R. (2019). General mental health is associated with gait asymmetry. Sensors.

[6] Potluri S., Ravuri S., Diedrich C., Schega L. Deep learning based gait abnormality detection using wearable sensor system. Proceedings of the 2019 41st Annual International Conference of the IEEE Engineering in Medicine and Biology Society (EMBC).

[7] Homayounfar S.Z., Andrew T.L. (2020). Wearable Sensors for Monitoring Human Motion: A Review on Mechanisms, Materials, and Challenges. SLAS Technol..

[8] Raknim P., Lan K.C. (2016). Gait Monitoring for Early Neurological Disorder Detection Using Sensors in a Smartphone: Validation and a Case Study of Parkinsonism. Telemed. E-Health.

[9] Altilio R., Rossetti A., Fang Q., Gu X., Panella M. (2021). A comparison of machine learning classifiers for smartphone-based gait analysis. Med. Biol. Eng. Comput..

[10] Gill S., Hearn J., Powell G., Scheme E. Design of a multi-sensor IoT-enabled assistive device for discrete and deployable gait monitoring. Proceedings of the 2017 IEEE Healthcare Innovations and Point of Care Technologies (HI-POCT).

[11] Fernandez I.G., Ahmad S.A., Wada C. (2020). Inertial sensor-based instrumented cane for real-time walking cane kinematics estimation. Sensors.

[12] Chamorro-Moriana G., Sevillano J.L., Ridao-Fernández C. (2016). A compact forearm crutch based on force sensors for aided gait: Reliability and validity. Sensors.

[13] Brull A., Zubizarreta A., Cabanes I., Rodriguez-Larrad A. (2020). Sensorized tip for monitoring people with multiple sclerosis that require assistive devices for walking. Sensors.

[14] Burgos C.P., Gartner L., Ballester M.A., Noailly J., Stocker F., Schonfelder M., Adams T., Tassani S. (2020). In-ear accelerometer-based sensor for gait classification. IEEE Sens. J..

[15] Najafi B., Khan T., Wrobel J. Laboratory in a box: Wearable sensors and its advantages for gait analysis. Proceedings of the 2011 Annual International Conference of the IEEE Engineering in Medicine and Biology Society.

[16] Chow J.W., Stokic D.S. (2021). Longitudinal Changes in Temporospatial Gait Characteristics during the First Year Post-Stroke. Brain Sci..

[17] Sun R., Moon Y., McGinnis R.S., Seagers K., Motl R.W., Sheth N., Wright J.A., Ghaffari R., Patel S., Sosnoff J.J. (2018). Assessment of postural sway in individuals with multiple sclerosis using a novel wearable inertial sensor. Digit. Biomark..

[18] Marin J., Marin J.J., Blanco T., de la Torre J., Salcedo I., Martitegui E. (2020). Is my patient improving? Individualized gait analysis in rehabilitation. Appl. Sci..

[19] Schlachetzki J.C., Barth J., Marxreiter F., Gossler J., Kohl Z., Reinfelder S., Gassner H., Aminian K., Eskofier B.M., Winkler J. (2017). Wearable sensors objectively measure gait parameters in Parkinson’s disease. PLoS ONE.

[20] Marxreiter F., Gaßner H., Borozdina O., Barth J., Kohl Z., Schlachetzki J.C., Thun-Hohenstein C., Volc D., Eskofier B.M., Winkler J. (2018). Sensor-based gait analysis of individualized improvement during apomorphine titration in Parkinson’s disease. J. Neurol..

[21] Cola G., Avvenuti M., Vecchio A., Yang G.Z., Lo B. (2015). An on-node processing approach for anomaly detection in gait. IEEE Sens. J..

[22] Figueiredo J., Santos C.P., Moreno J.C. (2018). Automatic recognition of gait patterns in human motor disorders using machine learning: A review. Med. Eng. Phys..

[23] Nassif A.B., Talib M.A., Nasir Q., Dakalbab F.M. (2021). Machine Learning for Anomaly Detection: A Systematic Review. IEEE Access.

[24] Stradiotti L., Perini L., Davis J. Semi-Supervised Isolation Forest for Anomaly Detection. Proceedings of the SIAM International Conference on Data Mining (SDM).

[25] Teufl W., Taetz B., Miezal M., Dindorf C., Fröhlich M., Trinler U., Hogan A., Bleser G. (2021). Automated detection and explainability of pathological gait patterns using a one-class support vector machine trained on inertial measurement unit based gait data. Clin. Biomech..

[26] Zhong X., Meng F.G., Zhang B., Yao J., Fan X.L., Zhao X.G. An OCSVM Based Assessment Method for Robotic Rehabilitation. Proceedings of the 2021 China Automation Congress (CAC).

[27] Otamendi J., Zubizarreta A., Portillo E. (2023). Machine learning-based gait anomaly detection using a sensorized tip: An individualized approach. Neural Comput. Appl..

[28] Gill S., Seth N., Scheme E. (2020). A multi-sensor cane can detect changes in gait caused by simulated gait abnormalities and walking terrains. Sensors.

[29] Mesanza A.B., Lucas S., Zubizarreta A., Cabanes I., Portillo E., Rodriguez-Larrad A. (2020). A Machine Learning Approach to Perform Physical Activity Classification Using a Sensorized Crutch Tip. IEEE Access.

[30] Delgado-Escano R., Castro F.M., Cozar J.R., Marin-Jimenez M.J., Guil N. (2019). An end-to-end multi-task and fusion CNN for inertial-based gait recognition. IEEE Access.

[31] Turner A., Hayes S. (2019). The Classification of Minor Gait Alterations Using Wearable Sensors and Deep Learning. IEEE Trans. Biomed. Eng..

[32] Khera P., Kumar N. (2020). Role of machine learning in gait analysis: A review. J. Med. Eng. Technol..

[33] Schmidl S., Wenig P., Papenbrock T. (2022). Anomaly detection in time series. Proc. VLDB Endow..

[34] Atallah L., Aziz O., Lo B., Yang G.Z. Detecting Walking Gait Impairment with an Ear-worn Sensor. Proceedings of the 2009 Sixth International Workshop on Wearable and Implantable Body Sensor Networks.

